# Clinical relevance of occult infections in spinal pseudarthrosis revision^[Fn d35e490]^

**DOI:** 10.1016/j.xnsj.2022.100172

**Published:** 2022-09-21

**Authors:** Marco D. Burkhard, Ali Hassanzadeh, Octavian Andronic, Tobias Götschi, Ilker Uçkay, Mazda Farshad

**Affiliations:** a University Spine Center, Department of Orthopedic Surgery; b Unit for Clinical and Applied Research; c Infectiology, Balgrist University Hospital, University of Zurich, Switzerland

**Keywords:** Spine, Spinal fusion, Revision surgery, Pseudarthrosis, Occult infection, Cutibacterium acnes

## Abstract

**Background:**

Occult infections in spinal pseudarthrosis revisions have been reported in the literature, but the relevance of such an infection on patient outcomes is unknown. We aimed to elucidate clinical outcomes and re-revision risks between patients with and without occult infections in spinal revision surgery for pseudarthrosis.

**Methods:**

In this matched case-control study, we identified 128 patients who underwent thoracolumbar revision surgery from 2014–2019 for pseudarthrosis of the spine. Among them, 13 (10.2%) revealed an occult infection (defined by at least two positive intraoperative tissue samples with the same pathogen), and nine of these 13 were available for follow-up. We selected 18 of the 115 controls using a 2:1 fuzzy matching based on fusion length and length of follow-up. The patients were followed up to assess subsequent re-revision surgeries and the following postoperative patient-reported outcome measures (PROMs): overall satisfaction, Oswestry Disability Index, 5-level EQ-5D, and Short Form 36.

**Results:**

Patient characteristics, surgical data, and length of follow-up were equal between both study groups. The rate of re-revision free survival after the initial pseudarthrosis revision surgery was higher in the occult infection group (77.8%) than the non-infectious controls (44.4%), although not significantly (0.22). The total number of re-revision surgeries, including re-re-revisions, was thirteen (in ten patients) in the control and two (in two patients) in the occult infection group (p = 0.08) after a median follow-up of 24 months (range 13-75). Four cases in the control group underwent re-revision for pseudarthrosis compared to none in the infected group. Satisfactory scores were recorded in all PROMs, with similar scores between the two groups.

**Conclusions:**

The presence of an occult infection accompanying spinal pseudarthrosis revision was not inferior to non-infected pseudarthrosis revisions in a matched, small sample size cohort study. This may be explained due to the possibility of targeted treatment of the identified cause of pseudarthrosis.

## Background

**Fig. 1:**
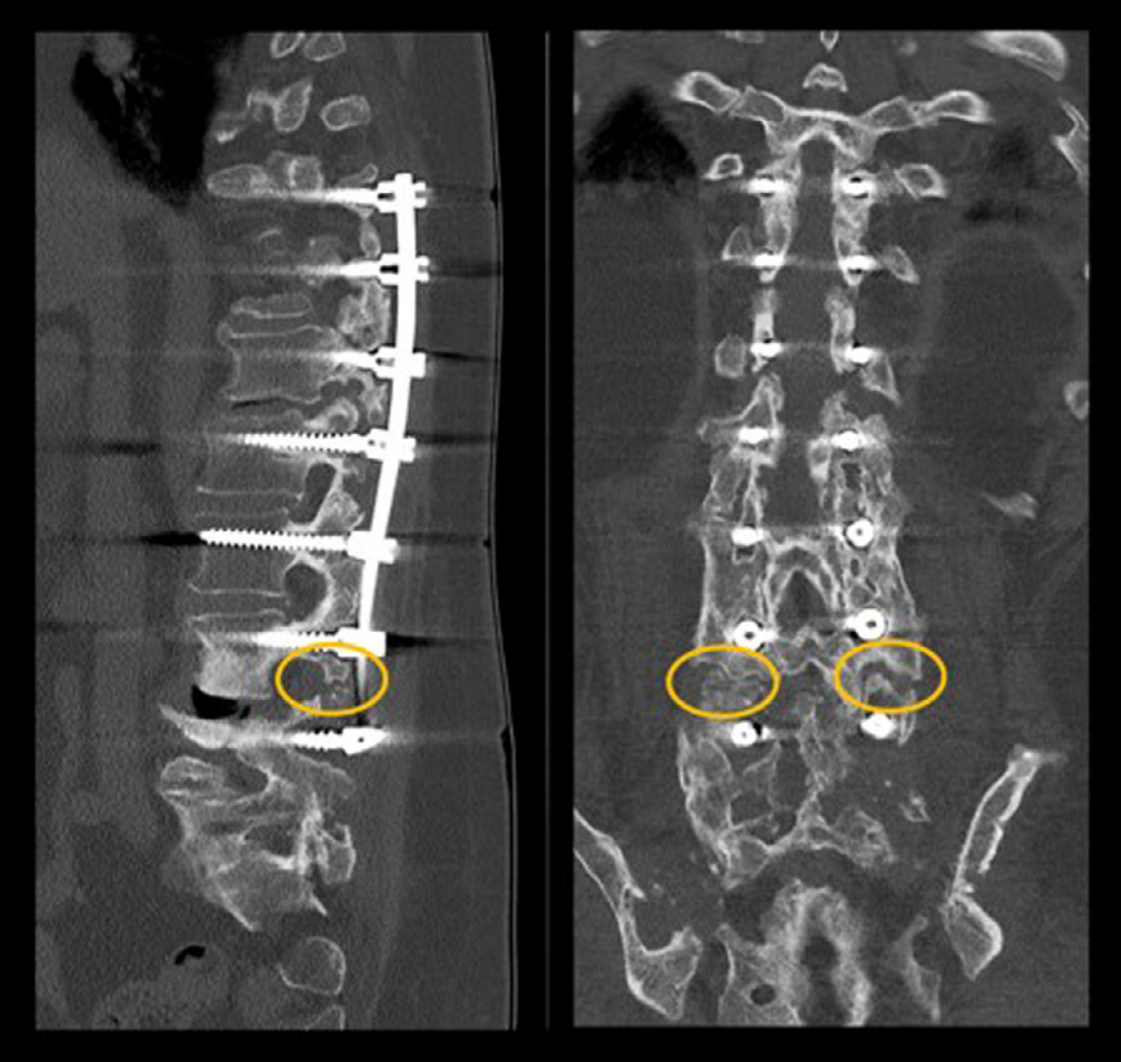
**Example of a pseudarthrosis case**. Lateral and coronar views of the computed tomography scan showing pseudarthroses at the level L4/5 beside large posterolateral fusion masses.

Pseudarthrosis, or failure of adequate bony fusion, is one of the most important and challenging reasons for revision surgery after attempted spinal fusion [1], [2], [3] (Figure 1). Besides insufficient primary implant construct stability; spinal imbalance, osteoporosis, tobacco and steroid use, infection has been identified as a major risk factor for pseudarthrosis [4], [5], [6], [7].

Clinicians frequently detect the presence of an occult low-grade infection during spinal revision surgery, with incidences varying between 9-56%, depending on the indication for revision and the method of tissue sampling [7], [8], [9], [10], [11], [12], [13], [14]. Pseudarthrosis is the leading indication for revision surgery associated with low-virulence bacteria [9,11]. In a previous study, we detected the presence of occult infections (defined as at least two positive samples of the same pathogen) in 10.2% of the pseudarthrosis cases not suspected to be infected [14]. Occult infection was associated with higher body mass index, fusions including the thoracolumbar junction, and slightly elevated C-reactive protein levels on admission [14]. In the literature, other patient- and surgery-related characteristics have been identified as risk factors for occult infection in spine surgery: male gender, older age, diabetes, higher American Society of Anesthesiologists' (ASA)-score, multiple comorbidities, higher number of prior surgeries [11,[15], [16], [17], [18]].

In general, long-term antibiotic treatment is recommended if an occult infection is detected, with the treatment-regimen being defined by interdisciplinary infectiology boards [14]. However, both clinicians and patients are often surprised by the detection of the occult infection, and the prognosis for these patients regarding risk for further re-revision surgery and patient satisfaction is unknown.

The aim of this study was to compare overall clinical outcomes and re-revision risks of patients undergoing pseudarthrosis revision surgery with and without occult infections.

## Methods

This study was approved by the state authorities (BASEC 2019-02077). Symptomatic spinal pseudarthrosis was defined as mechanical back pain related to failure of bony fusion or screw loosening on conventional radiographs and computed tomography (CT) at least > 6 months after the index surgery.

### Study population

We identified 152 adult patients who underwent pseudarthrosis revision surgery via a posterior-only approach since the introduction of routine sampling n pseudarthrosis cases in our institution in September 2014 until end of 2019 (Figure 1). After excluding 24 patients with an incomplete intraoperative microbiological sampling, we included 128 (84.2%) patients into the study. In none of the cases was an infection suspected and none of the patients had any antibiotic therapy prior to surgery, except of the preoperative prophylaxis. The initial surgery before the pseudarthrosis revision included a posterior pedicle screw instrumentation of an average of 3.4 ± 2.7 levels of attempted fusion for degenerative conditions in all patients. None of the patients had any antibiotic therapy prior to surgery except of the routine preoperative antibiotic prophylaxis and none of the patients had any prior surgical site infection diagnosed before the revision surgery. Beside pseudarthrosis and screw loosening on the radiologic examinations, there were no signs of inflammation preoperatively or intraoperatively such as wound healing disorders, redness, swelling, fluid formations, necrosis or implant corrosions.

Whereas almost half of the initial surgeries were performed outside, all revision surgeries were performed at our tertiary university spine department. We detected an occult infection in 13 (10.2%) patients, whilst 115 (89.8%) revealed negative samples. Four of these 13 infections were further excluded for the following reasons: two deaths (unrelated to surgery or infection) and two refusals to participate; leaving overall nine infected patients for follow-up assessments. None of the four excluded patients in the occult infection group underwent any further re-revision surgery after pseudarthrosis revision during their time of follow-up in our outpatient clinic (median 19 months, range 3-35 months).

**Fig. 2:**
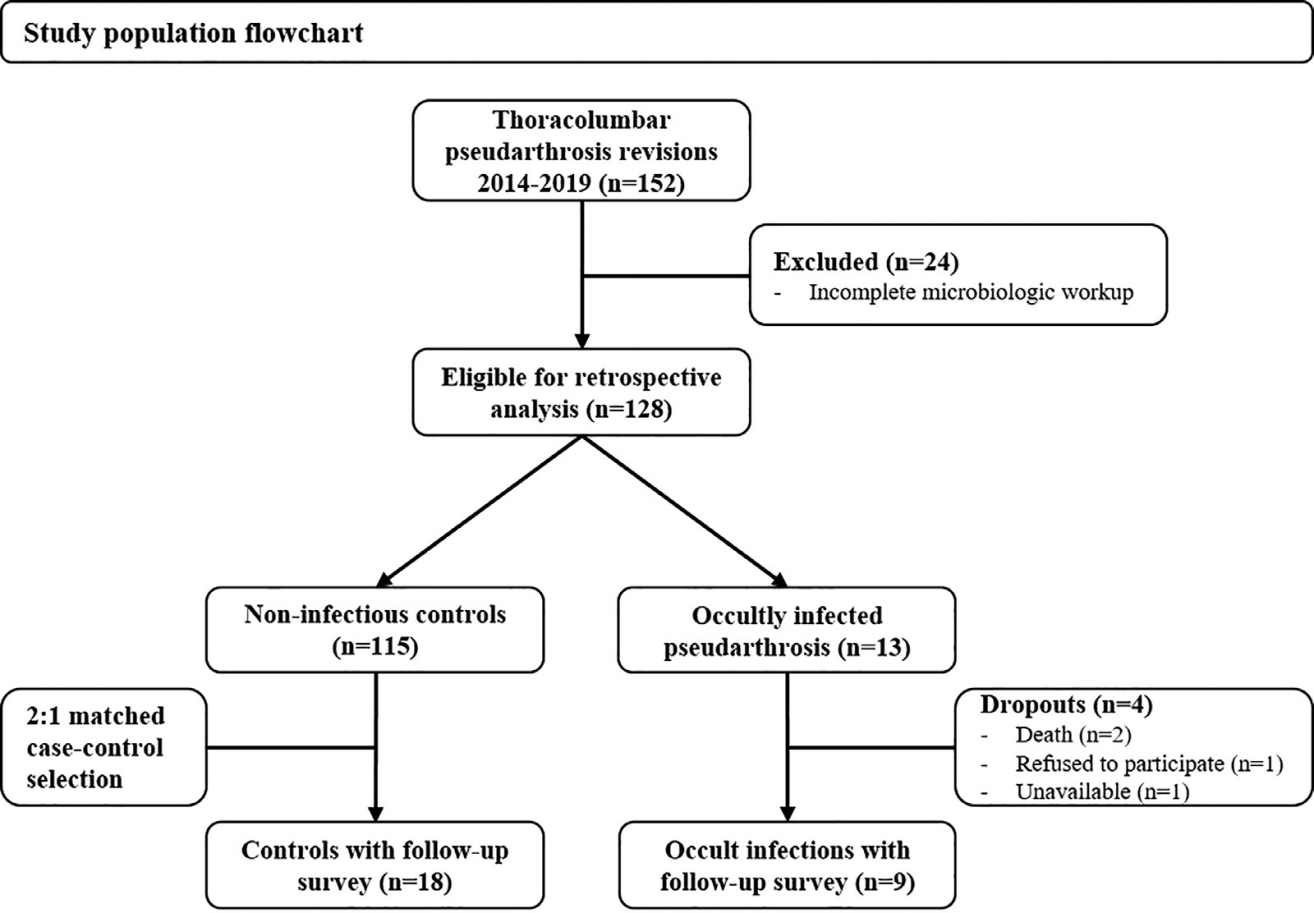
Study population flowchart.

As length of follow-up correlates with the risk of undergoing a revision operation and the length of fusion construct further impacts patient-reported outcome measures (PROMs), we matched the cohort using a 2:1 fuzzy matching based on these two factors. The fuzzy matching technique enabled the study population to forego any further sample size decrease in the group of nine occultly infected cases and roughly balance them (i.e. length of follow up +/- 6 months, length of fusion construct +/- 1 level) to the much larger pool of the non-infected controls (n=115). Thereby, we selected 18 out of the 115 uninfected patients as controls (figure 2). Besides the length of fusion and the length of follow-up, we recorded the following variables for the comparison of both study groups: age, sex, educational status, BMI, diabetes, ASA-score, prior number of lumbar surgeries and prior instrumentations involving the lumbosacral junction.

### Infections

We defined an occult infection as the presence of ≥ 2 intraoperative tissue samples that were incubated for 14 days for aerobic and anaerobic cultures. In general, two samples were gathered from the posterolateral pseudarthrosis and one from the lamina or pedicle, but additional samples were obtained from adjacent levels in some cases. Additionally, sonication of the removed hardware was routinely performed [19,20]. A pseudarthrosis was considered infected if 1) at least two tissue samples revealed the same pathogen and/or 2) at least 50 colonies of a bacterial species grew in the sonication fluid [21]. Perioperative antibiotic prophylaxis, mostly a second-generation cephalosporine (cefuroxime), was administered as part of the institutional standard protocol for spinal procedures

### Clinical outcomes

After a follow-up time ranging 13-75 months after revision surgery for pseudarthrosis of the spine, patients were asked about their general satisfaction regarding the clinical outcome (1 = poor, 2 = unsatisfactory, 3 = satisfactory, 4 = good, 5 = excellent). Details on any subsequent re-revision surgeries performed outside the index institution were recorded. The following PROMs were assessed: the Oswestry Disability Index (ODI), the 5-level version of the EuroQol five-dimensional self-administered questionnaire (EQ-5D-5L) and the Short Form 36 (SF-36). The summary index (SI) of the EQ-5D-5L was calculated based on the value set of the German population. Further, the EQ-5D visual analogue scale (VAS) was recorded, which measures health status on a vertical scale between 0 and 100 (worst-to-best imaginable health state) on the day of assessment. The eight scales of the SF-36 were also calculated based on the German population norm. Preoperative PROMs were not available for most patients and could not be included for analysis. All infected patients cured after recommended long-lasting antibiotic therapy.

### Statistical analyses

A fuzzy matching algorithm was performed using MATLAB *(*Matlab 2019a, MathWorks, Massachusetts, USA) to decrease the influence of confounding factors for patient outcomes. Fuzzy matching was performed on length of follow-up (+/- 6 months) and length of fusion (+/- 1 level). The 18 control patients were thereby matched to the nine occult infection cases. Intergroup analyses were performed using SPSS (version 25; IBM, Armonk, NY). As calculated per the Shapiro-Wilk test, data were non-normally distributed and data were reported using medians and ranges and ordinal variables were represented by frequencies (%). We used Mann-Whitney U-tests for continuous variables and the Fisher's exact tests for categorical variables. Due to the low number of infections, we renounced on multivariate analyses. The level of statistical significance was set at p < 0.05 for two-tailed analyses.

## Results

### Patients characteristics and infections

The demographics of the two study groups were similar (Table 1), other than age that tended to be higher in the controls (median 71 years) than their infected counterparts (median 58 years; *p*=0.06). In contrast, the number of prior lumbar surgeries was significantly lower in the controls (median 2 versus 3 interventions; p=0.027). None of the study patients smoked. The median follow-up time was 25 months (range, 13–74 mts) in the controls and 23 months (range, 15–75 mts) in the infection group, p=0.791.

**Table 1.** Patient characteristics.

Variable	Aseptic pseudarthrosis	Occult infection	*P* value
No. of patients	18	9	
Age (years)	71.5 (68; 75)	58 (55; 66)	0.057
Female sex (%)	38.9	33.3	1.000*
Education level (I-III)	I (11), II (61), III (28)	II (67), III (33)	1.000*
Body mass index (kg/m^2^)	29.7 (26.0; 31.7)	31.5 (27.8; 36.0)	0.304
Diabetes type II (%)	5.6	11.1	1.000*
Osteoporosis	11.1	11.1	1.000*
ASA	3 (2; 3)	3 (3; 3)	0.834*
Charlson Comorbidity Index	4 (3; 6)	2 (1; 3)	0.107
No. of prior lumbar surgeries	2 (1-4)	3 (1-7)	**0.027**
Follow-up (months)	25 (16; 61)	23 (19; 62)	0.719
No. of levels fused	4 (2; 6)	3 (2; 7)	0.856
Instrumentation involving L5/S1 (%)	77.8	77.8	1.000*

Data are given as median and 25^th^ and 75^th^ percentiles in parentheses. Categorical data are given in percentage. P values are calculated with Mann-Whitney U test or Fisher's exact test (marked with asterisk) as applicable. Bold text indicates statistical significance.

The diagnosis of symptomatic pseudarthrosis and screw loosening was made based on the CT findings in conjunction with excruciating pain and point tenderness over the pseudarthrosis segment in all 27 patients (9 infected and 18 controls). None of the subjects had an infectious etiology suspected by the clinician or radiologist pre-or intraoperatively. All patients were initially instrumented and revised from posterior only with pedicle-screw instrumentation, with or without transforaminal/posterior interbody fusion. *Cutibacterium acnes* was cultivated in five patients, *Staphylococcus epidermidis* in three patients, and *Enterococcus faecium* in one patient. There were no polymicrobial infections. No pathogens were isolated from any single probe in the control group, although at least 5 samples were taken per definition in any included pseudarthrosis revision.

Immediate targeted antibiotic treatment was initiated for three months for all infected patients according to the Infectious Diseases consultations at that time. *C. acnes* infections were treated with oral clindamycin. The antibiotic treatment of the staphylococci and of the *E. faecium* depended on the microbiologic profile, susceptibility testing, and patient's tolerance. None of the patients with an occult infection had any major complications related to the surgery or antibiotic treatment.

### Outcomes

During the time of follow-up, the rate of re-revision free survival after the initial pseudarthrosis revision surgery was higher in the occult infection group (77.8%) than the non-infectious controls (44.4%), although not significantly (0.22). The odds ratio for undergoing a re-revision surgery after pseudarthrosis revision was 0.4 (95% confidence interval 0.1 - 2.2) in the occult infection group versus the controls. The total number of re-revision surgeries, including possible re-re-revisions after the index pseudarthrosis revision, was thirteen (in ten patients) in the control and two (in two patients) in the occult infection group (p = 0.08) after a median follow-up of 24 months (range 13-75). Three patients in the control group each underwent two re-operations after the index pseudarthrosis revision during the time of follow-up. Four cases (22.2%) in the control group underwent re-revision surgery because of additional re-pseudarthrosis, whereas no pseudarthrosis re-revision was observed in the occult infection group.

The microbiological samples of the pseudarthrosis re-revisions (all without concomitant antibiotic influence) remained negative despite 14 days of incubation. Furthermore, three patients (16.7%) in the control group underwent more than one re-revision surgery following the initial pseudarthrosis revision. As for the infected group, only two patients underwent a further re-revision (one for screw-loosening and one for adjacent segment disease) and none of the two was further re-re-revised (Table 2).

**Table 2.** Revision rates and indications for revisions.

Variable	Aseptic pseudarthrosis	Occult infection	*P* value
No. of patients	18	9	
Re-revision free survival % (n)	44.4 (8)	77.8 (7)	0.217*
No. of revisions	1 (0; 1)	0 (0; 0)	0.083
Indication for revision % (n)			
Pseudarthrosis	22.2 (4)	0	0.268*
Screw loosening	11.1 (2)	11.1 (1)	1.000*
Adjacent segment disease	11.1 (2)	11.1 (1)	1.000*
Nerve root compression	11.1 (2)	0	0.536*

Data are given as median and 25^th^ and 75^th^ percentiles in parentheses. Categorical data are given in percentage. P values are calculated with Mann-Whitney U test or Fisher's exact test (marked with asterisk) as applicable.

Overall, we recorded satisfactory-to-poor scores in all PROMs, similarly distributed between the two study groups. Median patient satisfaction was 3 (satisfactory) in both groups (p=0.501). The ODI Score was 62 points (controls) versus 64 points (infected) (p=0.699). The EQ-5D-5L index was 0.31 in both groups (p=0.797), EQ-5D VAS was 58 (controls) versus 50 (infected) (p=0.365), and SF-36 scores were all similar in all the eight physical and mental scales. Further details are illustrated in Table 3.

**Table 3.** Patient reported outcome measures.

Variable	Aseptic pseudarthrosis	Occult infection	*P* value
No. of patients	18	9	
Patient satisfaction (1 – 5)	3 (2; 3)	3 (2; 3)	0.501
ODI			
Total	62 (56; 78)	64 (56; 76)	0.699
Pain	3 (2; 3)	3; 3 (1-4)	0.591
EQ-5D-5L			
Summary Index (-0.21 – 1)	0.31 (0.23; 0.51)	0.31 (0.24; 0.53)	0.797
EQ-5D VAS (0 – 100)	58 (50-74)	50 (30; 65)	0.365
SF-36			
Physical functioning	43 (25; 58)	30 (25; 50)	0.897
Bodily pain	62 (52; 74)	52 (52; 84)	0.675
Role limitations - physical health	0 (0; 50)	0 (0; 25)	0.929
Role limitations - personal/emotional	33 (0; 67)	100 (33; 100)	0.178
General mental health	76 (60; 80)	60 (44; 80)	0.326
Social functioning	38 (13; 63)	13 (0; 63)	0.896
Energy/fatigue or vitality	40 (30; 59)	35 (15; 55)	0.366
General health perceptions	57 (52; 69)	45 (37; 62)	0.155

ODI = Oswestry Disability Index. EQ-5D-5L = 5-level version of the EQ-5D tool. SF-36 = short form 36. Data are given as median and 25^th^ and 75^th^ percentiles in parentheses. P values are calculated with Mann-Whitney U test.

## Discussion

According to our single-center case-control study among adults who are surgically revised for spinal pseudarthrosis, the detection of an occult infection (even if relatively frequent) did not impair the patient's final outcome, provided that such infected pseudarthroses were surgically and antibiotically treated.

Because several patient and surgical history-related factors have been associated with less favorable outcomes after spinal fusion, we aimed to create two comparable groups and minimize confounding factors. However, despite the 2:1 fuzzy matching procedure, the two study groups still revealed some differences, most prominently the number of prior lumbar surgical procedures, which was significantly higher in the occult infection group. Also, a trend toward older age and a higher number of comorbidities was observed in the control group. Indeed, an increasing age and number of comorbidities are known to negatively impact clinical and/or radiographic outcomes after spinal fusion [22,23], but to be associated with slightly better outcomes in ODI scores compared to younger patients [24]. Likewise, Zehnder et al. [25] attributed worsening PROMs to the increasing the number of prior surgeries in degenerative lumbar spine surgery. Overall, the remaining differences between our two study cohorts were not in favor but rather at the expense of the occult infection group regarding expected outcome scores. Therefore, this methodological limitation would, if removed, even increase the evidence of the here reported non-inferiority.

All the investigated postoperative PROMs were at best satisfactory in both groups of this study. Although excruciating pain and point tenderness over the non-fused segment was reported preoperatively, it remains an unspecific and unreliable finding that only complements the clinical picture of a symptomatic pseudarthrosis and was not investigated postoperatively [26]. Some authors published significant subjective improvements after pseudarthrosis revision [5,27], whereas multiple others reported only modest to no improvements after revision surgery [28], [29], [30], [31], [32]. For example, Dede et al. reported that patients did not subjectively improve after pseudarthrosis revision [29]. Suh et al. found even worse outcomes after pseudarthrosis revision surgery than for adjacent segment disease [30]. On the other hand, many studies reported a correlation between bony fusion and satisfaction after spinal revision surgery, including for pseudarthrosis [33,34]. Kim et al. [34] reported a fusion rate of only 16 of 29 patients after pseudarthrosis revision, but 81% of the patients with successful fusion after pseudarthrosis revision had a satisfactory subjective outcome, compared to a satisfactory rate of only 23% in the unsuccessful fusions.

Our re-revision risks tended to be lower (22% versus 56%) in favor of the occult infection group, even though the difference was not statistically significant. In general, surgical site infections not only require enduring long-term antibiotic treatment, but are also associated with poorer clinical outcome scores and higher risk for revision surgery [35,36]. Therefore, we expected that the occultly infected patients would have lower PROMs together with higher re-revision risk when compared to their noninfected study group. The here reported counterintuitive observation may be explained by the fact that one main contributor to pseudarthrosis, namely an occult infection, was found in the case group and could be treated specifically both surgically and pharmaceutically. In contrast, the reasons for non-infectious pseudarthrosis development are generally multifactorial, and therapy of these patients may be less targeted. However, further large-scale studies are needed to clarify this observation.

In our occultly infected group, patients were infected with *C. acnes*, coagulase-negative staphylococci and *E. faecalis*. This reflects the most common spectrum of pathogens detected after spinal hardware removal for low-grade infection [14]. *C. acnes*, a slow-growing, microaerophilic Gram-positive rod, is usually considered as a contaminant by many authors [37], [38], [39], [40], [41], unless it is found in several intraoperative tissue samples. In such cases, and in the absence of concomitant, more virulent pathogens, *C. acnes* counts as the only and true pathogen causative of many spinal surgical site infections, spondylodiscitis, and vertebral osteomyelitis cases [3,42,43]. In our institution, routine tissue sampling and complete microbiological workup are performed in all spinal pseudarthrosis revisions. However, many surgeons still only obtain intraoperative samples when infection is visually suspected, which may lead to an underestimation of the true prevalence of infection-associated pseudarthrosis.

Some surgeons may argue that routine sampling in unsuspicious pseudarthrosis revisions will lead to too many false positives and unnecessary antibiotic treatments or that occult infections may be self-limiting after implant removal or replacement, and antibiotic treatment is redundant. From our personal standpoint, the risk of missing an infectious etiology, which is by evidence around 10% [14], and inadequate treatment by revisions surgery alone could outweigh the risk of antibiotic overtreatment of possible contaminations. Therefore, it is important that only the detection of the same bacteria in multiple samples qualifies for the diagnosis of an occult infection. In case of a delayed growth in only one enrichment broth, we consider the finding as a contamination and restrain from antibiotic treatment.

Besides the low sample size given by the rarity of the pathology and the obvious retrospective, observational design, our study has further limitations. Little is known about the performance of antibiotic treatment in low-grade infections. To our knowledge, no study has compared the outcomes of infected pseudarthrosis with revision surgery alone versus revision and antibiotic treatment. Finally, it is impossible to definitively distinguish between colonization and low-grade infection based on microbiological findings alone, even with the standardized workup protocol used in our institution. Two positive samples do not entirely rule out contamination of a pseudarthrosis which has been concomitantly caused by other reasons, but we define them as a true infection in concordance to previous studies [9,19]. Whereas many factors (i.e. age, gender, race, smoking status, bone quality and many more) are known to influence patient outcomes, the small sample size only allowed two factors (i.e. length of instrumentation and duration of follow-up) to be included in the 2:1 matching algorithm. Also, it is unclear if patients profited from the pseudarthrosis revision surgery, because preoperative PROMs were not available for analysis. Although excruciating pain and point tenderness was reported preoperatively, no postoperative data beside the PROMs questionnaires were included for analysis. With larger case numbers and statistical power, some of the observed effects may be substantiated. For example, the difference in rate of re-revision (22% in infected versus 56% in controls), which did not reach statistical significance, may be a type II error and may actually be a true difference in an adequately powered study analysis.

Based on our data, an a-priori sample size estimation using g*power (version 3.1.9.7) showed that a sample size of 39 in the occult infection group and 78 in the control group would be necessary to reach statistical significance (α < 0.05; power > 0.95) in a 2:1 matched study design. In an unmatched study assuming an occult infection rate of 10%, 30 infected spinal pseudarthrosis cases and 300 non-infected controls would be necessary to investigate whether re-revision rates truly differ in patients with occult infections. However, the rarity of the here investigated cohort, namely occult infected pseudarthrosis patients justifies reporting of this findings even if statistical underpowering might limit their evidence. Further, by plausibility, increase of sample size would tend to support the here reported non-inferiority of occult infection not impairing patient's final outcome, provided that such infected pseudarthroses are surgically and antibiotically treated.

## Conclusion

The presence of an occult infection accompanying spinal pseudarthrosis revision seem not to lead to inferior outcomes if compared to non-infected pseudarthrosis revisions in a matched, small sample size cohort study. This may be explained due to the possibility of targeted treatment of the identified cause of pseudarthrosis.

## Declaration of Competing Interest

The authors declare that they have no known competing financial interests or personal relationships that could have appeared to influence the work reported in this paper.
